# Molecular Cloning and Functional Analysis of the *NPR1* Homolog in Kiwifruit (*Actinidia eriantha*)

**DOI:** 10.3389/fpls.2020.551201

**Published:** 2020-09-16

**Authors:** Lei-Ming Sun, Jin-Bao Fang, Min Zhang, Xiu-Juan Qi, Miao-Miao Lin, Jin-Yong Chen

**Affiliations:** Zhengzhou Fruit Research Institute, Chinese Academy of Agricultural Sciences, Zhengzhou, China

**Keywords:** kiwifruit, NPR1, disease resistance, *Pseudomonas syringae* pv. a*ctinidiae*, systemic acquired resistance, salicylic acid

## Abstract

Kiwifruit bacterial canker, caused by the bacterial pathogen *Pseudomonas syringae* pv. *actinidiae* (Psa), is a destructive disease in the kiwifruit industry globally. Consequently, understanding the mechanism of defense against pathogens in kiwifruit could facilitate the development of effective novel protection strategies. The *Non-expressor of Pathogenesis-Related genes 1* (*NPR1*) is a critical component of the salicylic acid (SA)-dependent signaling pathway. Here, a novel kiwifruit *NPR1*-like gene, designated *AeNPR1a*, was isolated by using PCR and rapid amplification of cDNA ends techniques. The full-length cDNA consisted of 1952 base pairs with a 1,746-bp open-reading frame encoding a 582 amino acid protein. Homology analysis showed that the AeNPR1a protein is significantly similar to the VvNPR1 of grape. A 2.0 Kb 5′-flanking region of *AeNPR1a* was isolated, and sequence identification revealed the presence of several putative cis-regulatory elements, including basic elements, defense and stress response elements, and binding sites for WRKY transcription factors. Real-time quantitative PCR results demonstrated that *AeNPR1a* had different expression patterns in various tissues, and its transcription could be induced by phytohormone treatment and Psa inoculation. The yeast two-hybrid assay revealed that AeNPR1a interacts with AeTGA2. Constitutive expression of *AeNPR1a* induced the expression of pathogenesis-related gene in transgenic tobacco plants and enhanced tolerance to bacterial pathogens. In addition, *AeNPR1a* expression could restore basal resistance to *Pseudomonas syringae* pv. *tomato* DC3000 (Pst) in *Arabidopsis* *npr1-1* mutant. Our data suggest that *AeNPR1a* gene is likely to play a pivotal role in defense responses in kiwifruit.

## Introduction

Kiwifruit (*Actinidia* spp) is a perennial deciduous vine originating from China, and it is an emerging economic fruit tree. It has long been called “the king of VC” because of its unique flavor and high nutrient composition, being rich in vitamin C (Huang et al., 2013; Huang, 2016). Over the past few decades, kiwifruit has become an important horticultural crop, and numerous varieties have been commercialized. However, the majority of the currently cultivated *Actinidia chinensis* (i.e., Hort16A, Hongyang) and *Actinidia deliciosa* (i.e., Hayward) cultivars and their pollinators are highly susceptible to the infection of kiwifruit bacterial canker, a major kiwifruit disease caused by *Pseudomonas syringae* pv. *actinidiae* (Psa). The pathogen was first recognized and recorded in Japan in 1984 (Serizawa et al., 1989) and was subsequently described in Korea, Italy, and other kiwifruit production areas (Koh et al., 1994; Everett et al., 2011). Psa is the primary factor limiting kiwifruit production, and it is responsible for considerable economic losses in the kiwifruit industry globally. Therefore, a comprehensive understanding of kiwifruit disease defense mechanisms would facilitate the management and decrease of the effects of pathogen infection and the development of effective strategies for controlling Psa. In recent years, the genomes of several kiwifruit cultivars (i.e., *A. chinensis* Hongyang, Red5, and *A. eriantha* White) have been sequenced, which is convenient for the exploration of genes involved in defense responses and the study of regulatory mechanisms (Huang et al., 2013; Pilkington et al., 2018; Tang et al., 2019).

Plants have evolved complex regulatory networks of defenses to recognize and combat invading microbes and herbivorous insects, such as hypersensitive response (HR), systemic acquired resistance (SAR), and induced systemic resistance (ISR) (Kinkema et al., 2000). Such defense systems are tightly coordinated through the activities of several phytohormones, especially salicylic acid (SA). SA is a defense signal molecule that participates in resistance against biotrophic and hemibiotrophic pathogens by inducing SAR (Pieterse et al., 2009; An and Mou, 2011). SAR is one of the most commonly induced defense mechanisms and is typically triggered when plants are invaded locally by pathogens to provide long-lasting and broad-spectrum protection for the whole plant (Gao et al., 2015). The establishment of SAR involves the generation and transport of signals to uninfected distal tissues, which is highly dependent on the higher levels of *in vivo* SA, since blocking of SA accumulation inhibits SAR induction (Shine et al., 2019; Zhang and Li, 2019). When plants are attacked by a pathogen, SA concentrations increase from a basal level to a high level both locally and systemically. In addition, in many plants, exogenous application of SA or its functional analogs, such as 2, 6-dichloroisonicotinic acid (INA) and benzo (1,2,3) thiadiazole-7-carbothioic acid S-methylester (BTH), can also induce SAR and enhance plant resistance to disease (Görlach et al., 1996; Wu et al., 2012).

Despite the underlying mechanisms of the relationship between SA and SAR have not yet been elucidated comprehensively, Extensive studies in model plant *Arabidopsis* have shown that the Non-expressor of Pathogenesis-Related genes 1 (NPR1), also known as Non-inducible immunity 1 (NIM1) or Salicylic acid insensitive 1 (SAI1), played a crucial role in salicylic acid-mediated SAR (Loake and Grant, 2007; Chen et al., 2019). NPR1 belongs to a small NPR1-like family that contains four NPR1homologs (AtNPR1-AtNPR4) and two BOP homologs (AtBOP1 and AtBOP2) in *Arabidopsis*. Phylogenetic analysis of this family revealed three functionally distinct clades (Zhang et al., 2006; Kuai and Després, 2016). The most widely studied member of the *NPR1*-like family is the *NPR1* gene. *NPR1* was originally identified in a screen of *Arabidopsis* mutants that were unable to express the *PR* genes during pathogen invasion (Cao et al., 1994). Mutant *npr1* plants not only compromised SAR response in the presence of the SA or SA analogs, exhibiting little expression of *PR* genes, but also increased susceptibility to pathogens (Cao et al., 1997). The *Arabidopsis NPR1* (*AtNPR1*) gene encodes a protein with a BTB/POZ (Broad-complex, Tramtrack and Bric-a-brac/Pox virus, and Zinc finger) domain and an ankyrin repeats domain, both of which mediate protein-protein interactions (Cao et al., 1997; Sandhu et al., 2009; Backer et al., 2019). It also possesses several functional amino acid residues, such as Cys^82^, Cys^150^, Cys^155^, Cys^160^, and Cys^216^, which are crucial for oligomer-monomer transition. Cys^156^ facilitates NPR1 oligomerization *in vivo via* S-nitrosylation, while Arg^432^ in the C-terminus serve for SA binding (Mou et al., 2003; Tada et al., 2008; Ding et al., 2018; Wang et al., 2020). In *Arabidopsis*, the *NPR1* is expressed constitutively at low levels and encodes an oligomerized, cytosolic protein. During pathogenic infection or SA treatment, the NPR1 oligomer in the cytoplasm disintegrates into its monomeric form, which translocates into the nucleus to interact with several TGA family members, and subsequently induces the transcription of *PR* genes (Kinkema et al., 2000; Fan and Dong, 2002). The redox modification and subsequent translocation to the nucleus of NPR1 are both necessary for SAR (Mou et al., 2003).

It has been confirmed that *NPR1* functions as the central regulator in plant defense networks, including the cross-talk between SA-JA defense pathways and the jasmonic acid (JA)/ethylene (ET)-dependent induced responses (Spoel et al., 2003; Leon-Reyes et al., 2009; Pieterse et al., 2009). The over-expression of *AtNPR1* transcript in *Arabidopsis* plants led to enhanced resistance to both bacterial and fungal pathogens, such as *Pseudomonas syringae*, *Peronospora parasitica*, and *Hyaloperonospora arabidopsidis*, in a dosage-dependent manner (Cao et al., 1998; Friedrich et al., 2001). Similarly, ectopic expression of *AtNPR1* in other crops including rice, wheat, tomato, soybean, apple, strawberry, citrus, and olive also resulted in resistance to a wide range of pathogens (Chern et al., 2001; Lin et al., 2004; Makandar et al., 2006; Malnoy et al., 2007; Matthews et al., 2014; Dutt et al., 2015; Silva et al., 2015; Robertson et al., 2018; Narváez et al., 2020). In transgenic cotton and tobacco plants, over-expression of *AtNPR1* conferred resistance to *Rotylenchulus reniformis* and *Spodoptera litura*, respectively (Meur et al., 2008; Parkhi et al., 2010). Moreover, homologs of *AtNPR1* have been isolated and characterized in several economically important crops. For example, constitutive expression of the apple homolog of *NPR1*, *MhNPR1*, resulted in the activation of *PR* genes and enhanced resistance against *Podosphaera leucotricha* in transgenic apple (Chen et al., 2012). In addition, *OsNPR1/NH1* overexpression in rice conferred resistance to bacterial blight, while silencing of the gene exhibited enhanced susceptibility to the pathogen (Yuan et al., 2007; Bai et al., 2011). Most recently, mulberry *MuNPR1* overexpression in transgenic *Arabidopsis* enhanced resistance against Pst and inhibited callose deposition (Xu et al., 2019). In periwinkle, silencing of *CrNPR1* significantly repressed *CrPR1a* induction and accelerated periwinkle leaf yellowing symptom development following *Tobacco rattle virus* (TRV) infection (Sung et al., 2019). The results of the studies above suggest that similar defense mechanisms exist across many crop species, and manipulating the expression of *NPR1* or its ortholog could be an effective strategy of improving crop disease resistance (Malnoy et al., 2007; Yuan et al., 2007; Silva et al., 2018).

In the present study, we isolated and characterized a novel full-length *NPR1* homolog, designated *AeNPR1a*, from a kiwifruit (*A. eriantha*) with high resistance to Psa. The sub-cellular localization of AeNPR1a based on the expression of GFP fusion proteins in *Arabidopsis* protoplasts indicated its presence in nuclei. Analysis of the 5′-upstream region identified potential cis-elements responsible for the regulation of the expression of *AeNPR1a*. Subsequently, we investigated the tissue expression levels of the gene and its expression profiles following treatment with SA, methyl jasmonate (MeJA), and Psa using RT-qPCR. Interaction between AeNPR1a and AeTGA2 was assayed using the yeast two-hybrid system. The effect of *AeNPR1a* over-expression on the growth of pathogenic bacteria in transgenic plants was also explored.

## Materials and Methods

### Plant Materials

The plant material used in the present study is *A. eriantha*, and plants were grown at the Kiwifruit Germplasm Repository of the National Horticulture Germplasm Resources Center in Zhengzhou, Henan province, China (N 34°42′, E 113°41′, 106 m). Several 4-year-old *A. eriantha* plants were selected for the collection of samples for tissue-specific expression analysis. All tissues were immediately frozen with liquid nitrogen and stored at −80°C until their subsequent use. Each biological sample was collected from three individual plants in the present study.

### Cloning of Full-Length AeNPR1a cDNA and Genomic DNA

Total RNA for cDNA synthesis was extracted from kiwifruit leaves using a modified CTAB method (Japelaghi et al., 2011). The RNA samples were treated with 2 U DNase I (Takara, Dalian, China) for 30 min at 37°C and then purified further. The first-strand cDNA was synthesized using approximately 1 μg of total RNA and the Oligo d(T)_18_ adaptor primer by RevertAid First Strand cDNA Synthesis Kit (Thermo Scientific, Waltham, MA, USA) according to the manufacturer’s instructions.

To clone the full-length cDNA of the *NPR1*-like gene from *A. eriantha*, two pairs of primers (**Supplementary Table S1**) were designed based on the partial coding sequence of *AeNPR1a*, which has been annotated in our previous RNA-seq data. The 5′ and 3′ rapid amplification of cDNA ends (RACE) strategies were performed using the SMART™ RACE cDNA Amplification Kit (Clontech, Mountain View, CA, USA) according to the manufacturer’s instruction. Thus, a pair of gene-specific primers, NPR1-01 and NPR1-02 (**Supplementary Table S1**), was designed based on the 5′-UTR and 3′-UTR for full-length cDNA. The PCR products were purified using a DNA Gel Purification Kit (GENEray, Shanghai, China), subcloned into the pMD18-T vector (Takara, Dalian, China), and at least three clones were selected for sequencing. The sequence was uploaded to the GenBank database of National Center of Biotechnology Information (NCBI; accession no. MN544777). Furthermore, the DNA from young leaves was extracted using a genomic DNA purification kit (Promega, Madison, WI, USA) and treated with RNase I (Takara, Dalian, China). *AeNPR1a* gene cloning from genomic DNA was also performed based on the full-length cDNA, and then, the target sequence was purified and sequenced as above.

### Sequence Alignment and Phylogenetic Analysis

The amino acid sequence of AeNPR1a was predicted using NCBI Open Reading Frame (ORF) finder, and was then submitted for analysis by SMART (Letunic and Bork, 2018). The BLAST search program was used for gene sequence similarity searches in NCBI (https://blast.ncbi.nlm.nih.gov/Blast.cgi). The exon/intron organization of *NPR1* genes was generated using the Gene Structure Display Server 2.0 (http://gsds.cbi.pku.edu.cn/). For protein sequence analysis, the coding sequence of *AcNPR1a* was also isolated from *Actinidia chinensis* based on the *AeNPR1a* and genomic information. Sequences with high homology to AeNPR1a and AcNPR1a were aligned using Clustal W (https://www.ebi.ac.uk/Tools). To identify NPR1-like proteins in kiwifruit, sequence of the known *Arabidopsis thaliana* NPR1-like proteins were queried against *A. chinensis* and *A. eriantha* genomic databases (Pilkington et al., 2018; Tang et al., 2019) using BLAST. Subsequently, phylogenetic analysis and phylogenetic tree construction were performed using the Maximum Likelihood method in MEGA 5 (Tamura et al., 2011), with bootstrap confidence values from 1,000 replicates.

### Isolation and Bioinformatics Analysis of AeNPR1a Promoter

To obtain the 5′-flanking region of *AeNPR1a*, genomic DNA was digested with EcoRV, DraI, StuI, and PvuII (TaKaRa, Dalian, China). Then, the digested DNA were purified and ligated separately with Genome Walker adaptors using the BD Universal Genome Walker™ kit (Clontech, Mountain View, CA, USA) according to the manufacturer′s instructions. Nested PCR was carried out using the prepared DNA as a template with the adaptor primers and gene specific primers (**Supplementary Table S1**). The final purified PCR products were cloned into pMD18-T vector (TaKaRa, Dalian, China) and sequenced. To identify the putative regulatory motifs of *AeNPR1a* promoter, bioinformatic analyses were carried out using PLACE (Higo et al., 1999) and PlantCARE (Lescot et al., 2002) databases.

### Subcellular Localization Assay

The full-length open reading frame (ORF) of *AeNPR1a* without the termination codon was cloned into the pBI121-EGFP vector to generate a fusion construct under the control of the cauliflower mosaic virus (CaMV) 35S promoter. The empty vector was used as a control. After sequence confirmation, these recombinant plasmids were transformed into *Arabidopsis* mesophyll protoplasts and onion epidermal cells as previously described (Yoo et al., 2007). Nuclei were stained with 50 μg·ml^−1^ 4′,6-diamidino-2-phenylindole (DAPI; Sigma, St. Louis, MO, USA) after a 24-h incubation, and then, the GFP fluorescence signal was observed and imaged with a laser confocal microscope (Zeiss, Oberkochen, Germany). The excitation lines were configured at 405 and 488 nm.

### Treatment of A. eriantha With SA, MeJA, and Psa Pathogen

To further investigate the induction mechanism of *AeNPR1a*, the spring shoots of 2-year-old plants were sprayed with SA (5 mM) (Sigma, St. Louis, MO, USA) and MeJA (0.1 mM) (Sigma, St. Louis, MO, USA), separately. Control plants for each treatment were treated with sterile distilled water containing equal amounts of the solvent (0.1% ethanol v/v) used for hormone preparation. Leaf samples were harvested from control and hormone-treated plants after 0, 6, 12, 24, 48, 72, and 96 h for RNA isolation. For bacterial infection, Psa-2 (Biovar 3) was grown on King’s B agar medium at 25°C for 2 days. Subsequently, a single colony was inoculated in liquid King’s B medium and incubated on a shaker for 16 to 20 h at 25°C until the OD_600_ reached 0.6. The bacterial cells were collected by centrifugation at 4000×g for 10 min and diluted to the desired concentration (10^6^ colony forming units (CFU)·ml^−1^) with 10 mM MgCl_2_ for plant inoculation. The bacterial suspension was infiltrated on the abaxial surface of 2-year-old plant leaves with sterile syringes, and then, the inoculated plants were kept in a growth chamber at 80% relative humidity and 25°C. Control plants were mock-inoculated with 10 mM MgCl_2_ and incubated separately to prevent cross-contamination. Leaf samples from the control and bacteria inoculated plants were harvested at different time points and immediately frozen in liquid nitrogen and stored at −80°C. All the treatments had three replicates.

### Gene Expression Analysis by Real-Time Quantitative RT-PCR

The spatio-temporal expression patterns of *AeNPR1a* in different tissues and its expression profiles in treated and control leaves were tested by quantitative real-time RT-PCR. RNA extraction and cDNA synthesis were carried out as described above.

For quantitative RT-PCR analysis, cDNA was diluted with RNase-free water before use. Real-time PCR of target genes was performed with specific primers using a LightCycler™ 480 Real-time PCR system with 2×SYBR Green Master Mix (Roche Applied Science, Mannheim, Germany). Kiwifruit *β-actin* was used as the internal reference gene. Primer sequences are listed in **Supplementary Table S1**. Real-time PCR products were amplified with 1-μl template of the RT reaction mixture, 10-μl 2×SYBR Green Master Mix, 0.5 μl each forward and reverse primer (10 μmol·μl^−1^), and water, with a final volume of 20 μl. The qRT-PCR amplification conditions were 95°C for 5 min, and then, 40 cycles of 95°C for 10 s, 60°C for 30 s, and 72°C for 15 s. Data were evaluated by calibrator-normalized relative quantification with efficiency correction using the RelQuant software version 1.01 or the LightCycler™ 480 software version 1.5 (Roche Applied Science, Mannheim, Germany) according to the 2^—△△CT^ method. Three biological replicates and four technical replicates were assayed.

### Yeast Two-Hybrid Assays

The experimental procedures of yeast two-hybrid assays were performed using the BD Matchmaker system (Clontech, Mountain View, CA, USA). The entire ORF and different regions from *AeNPR1a* were digested using Nde I (Takara, Dalian, China) and BamH I (Takara, Dalian, China) and subcloned into pGBKT7 vector, and the ORF of *AeTGA2* was cloned into the pGADT7 vector. All constructs were transformed into AH109 competent yeast cells according to the manufacturer′s instruction. The co-transformed yeast clones were also streaked on selective medium without tryptophan, histidine, leucine, and adenine, but supplemented with 20 μg·ml^−1^ 5-bromo-4-chloro-3-indoxyl-α-D-galactopyranoside (X-α-Gal) (Sigma, St. Louis, MO, USA) to further confirm positive interactions.

### Construction of Expression Vector and Genetic Transformation

To produce a vector for the constitutive expression of *AeNPR1a*, Bgl II (Takara, Dalian, China) and BstE II (Takara, Dalian, China) sites were introduced at the end of the full-length cDNA sequence of *AeNPR1a* by PCR amplification, and then, the fragment was digested and subcloned into the pCAMBIA 1301 vector (CAMBIA, Canberra, Australia) driven by CaMV35S promoter. The resulting recombinant plasmid was sequenced to verify the absence of PCR errors.

For plant transformation, the *35S::AeNPR1a* vector was transferred into *Agrobacterium tumefaciens* strain EHA105 using the freeze-thawing method (Holsters et al., 1978). Transformation of tobacco (*Nicotiana tabacum* cv. NC89) was performed using the leaf disc co-cultivation method as described by Krügel et al. (Krügel et al., 2002). The transgenic seedlings were regenerated under 10 mg·L^-1^ hygromycin B selection, transferred to soil, and grown in a growth chamber at 23 ± 1°C under 16h/8h light/dark conditions. To investigate whether the *AeNPR1a* gene was homologous to *Arabidopsis NPR1*, the *35S::AeNPR1a* vector was also transformed into the *Arabidopsis npr1-1* mutant *via* the floral dip method (Clough and Bent, 1998). Transgenic plants of the T_2_ or T_3_ generation and single-copy lines were used in further analyses.

### Pathogen Challenge of Transgenic Plants

*AeNPR1a* transgenic lines were evaluated for disease resistance to both Pst and Psa pathogens as previously described with a few modifications (Liu et al., 2005). Briefly, the strains were re-suspended to 10^6^ CFU· ml^−1^ in 10 mM MgCl_2_ (Sigma, St. Louis, MO, USA) solution. The leaves of 4-week-old transgenic and non-transgenic tobacco were inoculated by infiltration with 50-μl bacterial suspensions for Pst and Psa resistance evaluation, and infection of *Arabidopsis* was performed on 4-week-old *npr1*, wild-type, and transgenic plants by spraying for Pst resistance evaluation. The inoculated plants were placed in a growth chamber under 16-h/8-h light/dark conditions with 80% relative humidity at a constant temperature of 25°C. Three days after inoculation, leaf punches were collected from treated leaves, surface sterilized, and ground in 1 ml sterile 10 mM MgCl_2_. The suspensions were serially diluted and plated on King’s B agar medium. After 2 days of incubation at 25°C, |the average number of CFU per leaf disk was calculated and statistically analyzed. For phenotypic observation, the leaves of the control mock plants (inoculated with 10 mM MgCl_2_) and pathogen-inoculated plants were photographed 7 days after infection. The inoculation of each strain was repeated three times, and mean values and standard deviations were obtained with three leaves from three independent plants.

### Statistical Analysis

Statistical analysis was carried out using the SPSS statistics program (SPSS Inc., Chicago, USA). Data were analyzed using Student’s t-test for pairwise comparisons and Duncan’s multiple range test for multiple comparisons. Significant differences were evaluated at P < 0.05.

## Results

### Amino Acid Sequence Alignment and Phylogenetic Analysis of AeNPR1a

**Figure 1:**
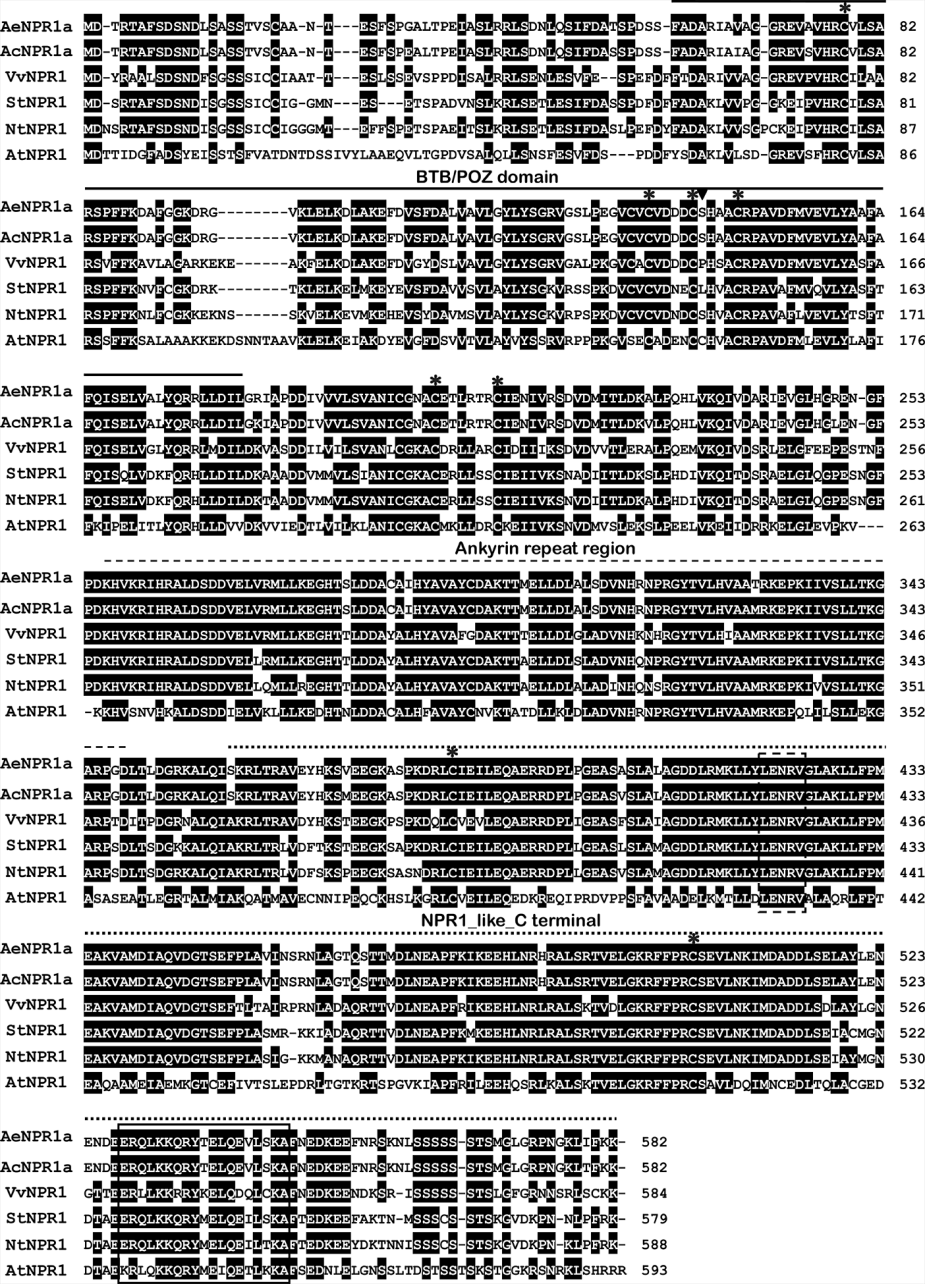
Multiple alignment of AeNPR1a protein with other NPR1 homologs. The BTB domain, Ankyrin repeat region and NPR1_like_C terminal are indicated with solid lines, broken lines and dotted lines, respectively. The conserved cysteine residues among the proteins are labeled by asterisks. The Cys^156^ relative to *Arabidopsis* is marked by inverted triangle. The putative nuclear localization signal (NLS) and LENRV are highlighted by solid line box and dashed line box, respectively. AcNPR1a (PSS20797), VvNPR1 (XP_002281475), StNPR1 (XP_006357709), NtNPR1 (AF480488), and AtNPR1 (AT1G64280).

**Figure 2:**
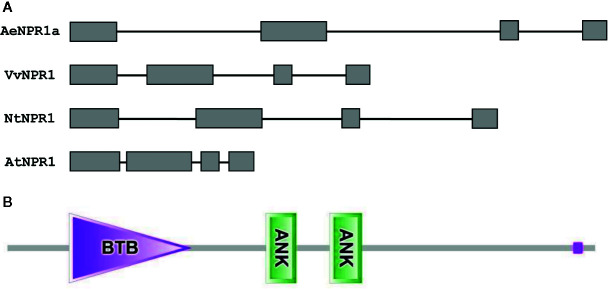
Analysis of *AeNPR1a* gene and protein structure. **(A)** Comparison of the exon-intron structures of *AeNPR1a* gene homologs. Exons and introns are represented by boxes and black lines, respectively. **(B)** The conserved domains of the AeNPR1a protein after analyzed by SMART.

The full length sequence of *AeNPR1a* (GenBank accession no. MN544777) was isolated from *A. eriantha* by the RACE method. The sequence was identical to the corresponding gene in the published sequenced genome. The open reading frame of *AeNPR1a* encoded a deduced 582 amino acid protein of 64.26 kDa, and the predicted isoelectric point (pI) was 5.82. Alignment of the deduced amino acid sequence revealed that AeNPR1a is most closely related to AcNPR1a (98.11% identity), which originates from the same genus, with only 10 residues varying, followed by VvNPR1 (74.06% identity), StNPR1 (73.21% identity), NtNPR1 (71.94% identity), and AtNPR1 (52.86% identity) (**Figure 1**). The genomic structure of *AeNPR1a* was similar to that of the other *NPR1* orthologs, sharing four exons and three introns (**Figure 2A**). Protein sequence comparison showed that AeNPR1a contained two conserved domains, a BTB/POZ domain and an ankyrin repeat domain (**Figures 1**, **2B**). Eight highly conserved cysteine residues were identified in AeNPR1a, which contained the five Cys residues that are required for oligomer-monomer transition of AtNPR1. However, functionally relevant residues Cys^156^, described for oligomerization, are not present in AeNPR1a (**Figure 1**). Nonetheless, a putative nuclear localization signal (NLS) and a penta-amino acid motif (LENRV) were present in the C-terminal of AeNPR1a (**Figure 1**).

**Figure 3:**
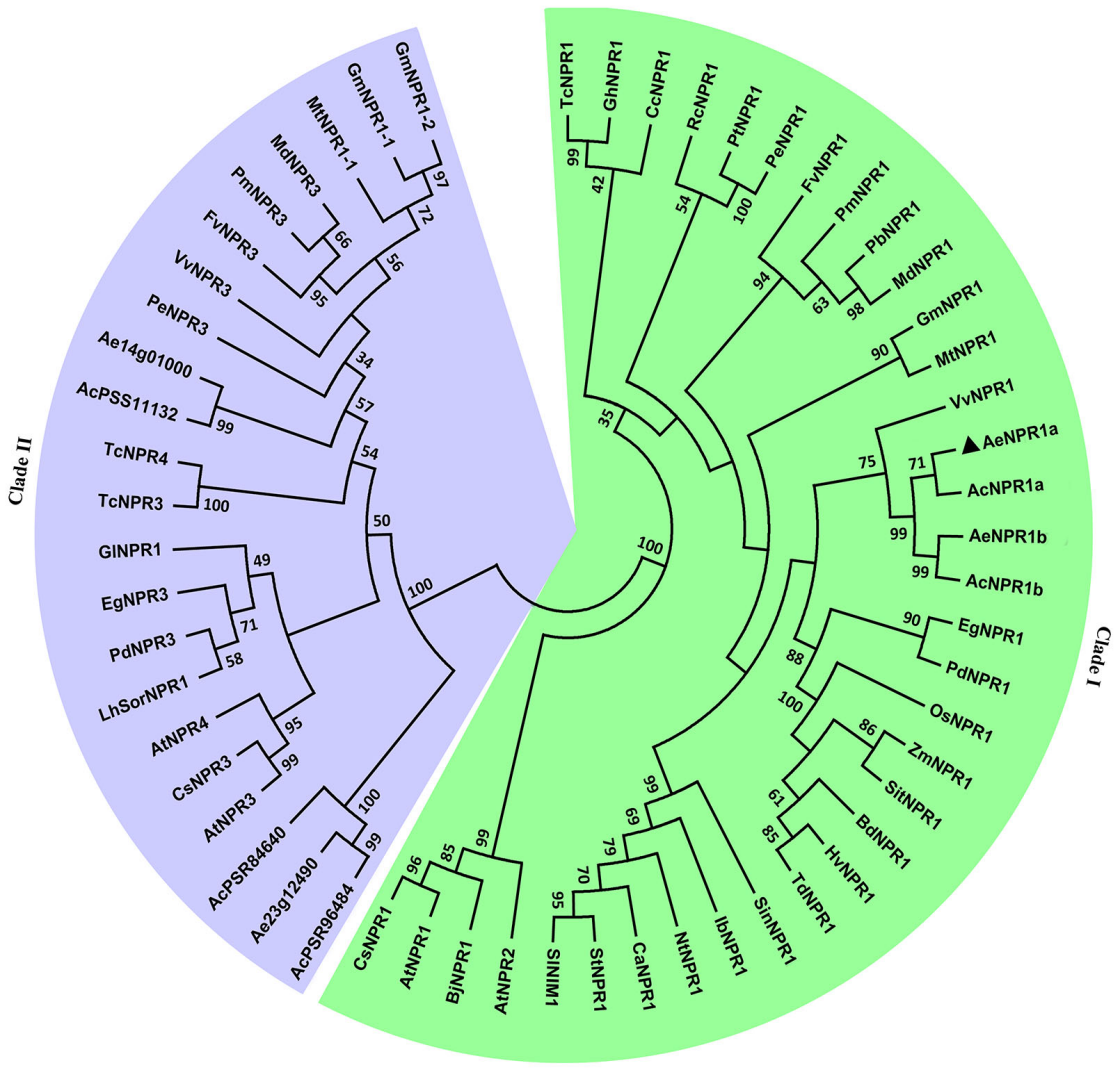
Phylogenetic relationship of AeNPR1a and other NPR1 homologs from different plant species. The tree was generated using the Maximum Likelihood method with 1,000 bootstrap replicates. AeNPR1a is indicated by a triangle. The accession numbers and species names for the NPR1 homolog sequences used in the analysis were summarized in **Supplementary Table S2**.

To reveal the evolutionary relationship between AeNPR1a and other NPR1-like proteins from various plant species, the AeNPR1a and AeNPR1a-like protein sequences obtained in the present study were subjected to phylogenetic analysis with other NPR1 homologs. As shown in **Figure 3**, the tree was grouped into two major clades, AeNPR1a was the most closely related to AcNPR1a, AcNPR1b, AeNPR1b, as well as VvNPR1, clustering with the clade containing AtNPR1 and AtNPR2. The other five members from *A. Chinensis* and *A. eriantha* were grouped into clade II along with AtNPR3 and AtNPR4. The data suggest that *AeNPR1a* may be a homolog of *NPR1* in kiwifruit.

### Subcellular Localization of AeNPR1a

**Figure 4:**
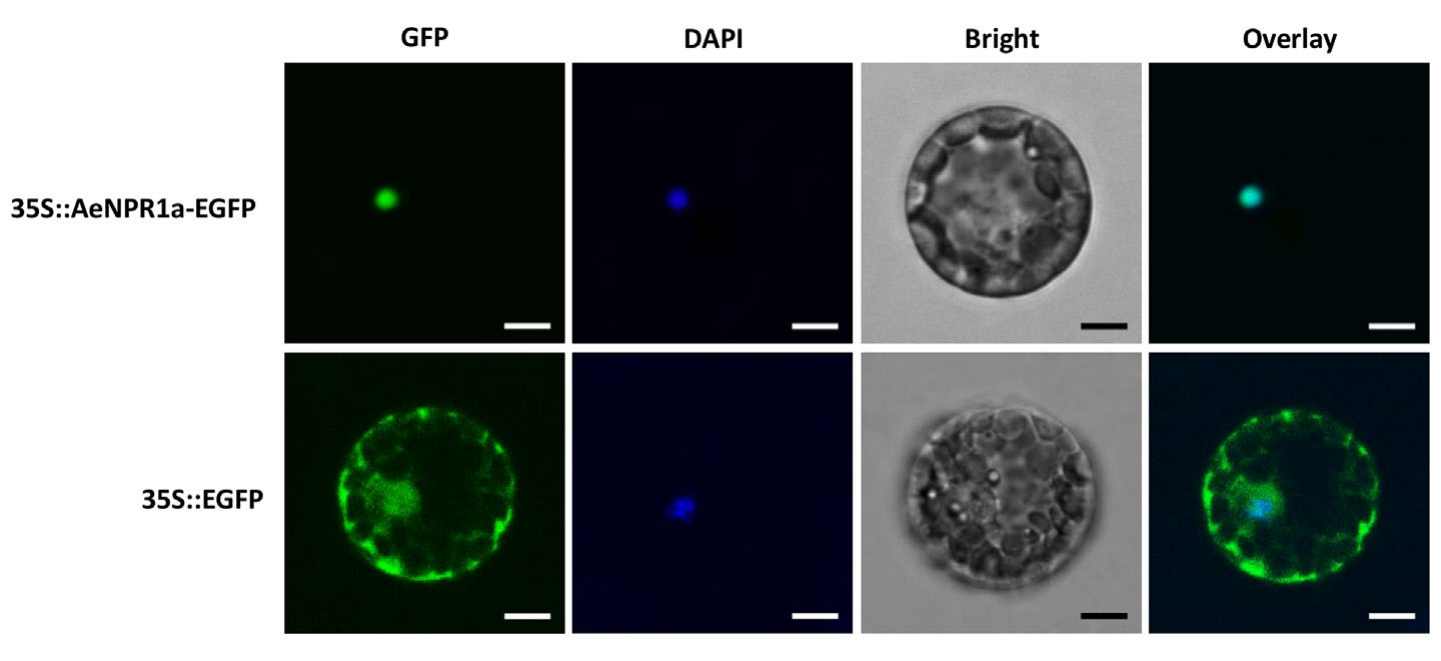
Subcellular localization of AeNPR1a protein. The constructs of *35S::AeNPR1a-EGFP* and *35S::EGFP* were transformed into *Arabidopsis* mesophyll protoplast cells by PEG-mediated transformation. Fluorescence signals were examined by a laser confocal scanning microscope. Nuclei of the cells were visualized DAPI staining. Overlay: merged GFP and DAPI images. Scale bars = 5 μm.

To examine the subcellular localization of AeNPR1a, the coding region of *AeNPR1a* without the stop codon was fused to the 5′ end of the enhanced green fluorescent protein (EGFP) reporter gene under the control of CaMV35S promoter. Subsequently, the fusion construct *35S::AeNPR1a-EGFP* was introduced into *Arabidopsis* mesophyll protoplasts and onion epidermal cells. GFP fluorescence was observed under a confocal fluorescent microscope. The results indicated that the fluorescence of AeNPR1a-EGFP was predominantly detected in the nucleus (**Figure 4** and **Supplementary Figure S1**). In contrast, the control vector expressing free EGFP yielded fluorescence in both the nucleus and cytoplasm (**Figure 4** and **Supplementary Figure S1**). Therefore, AeNPR1a appeared to be primarily a nuclear protein in these cell types.

### Characterization of Potential Cis-Acting Elements in the AeNPR1a Promoter

**Figure 5:**
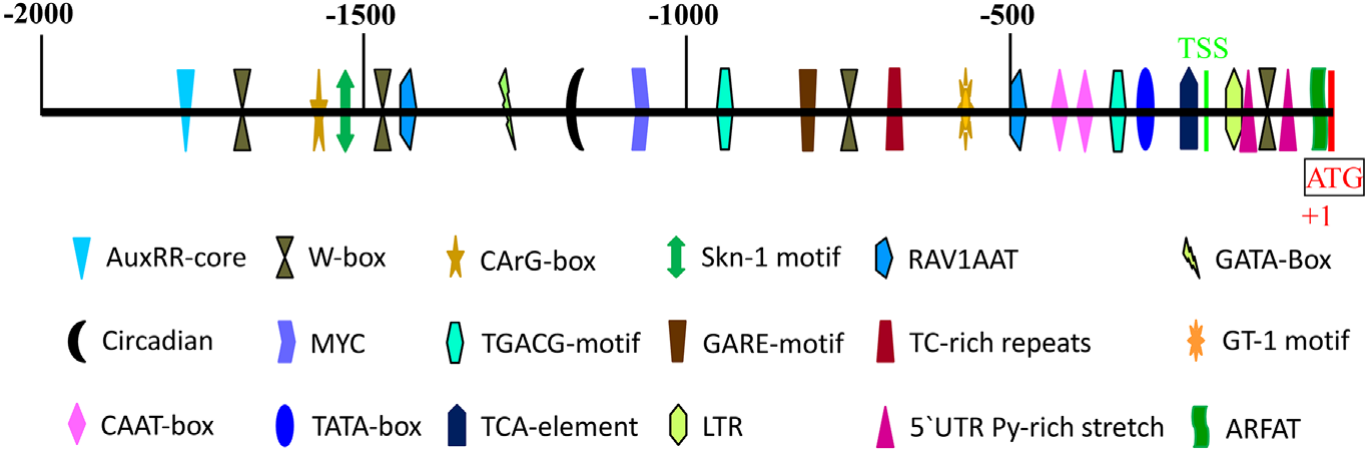
Schematic representation of predicted cis-acting regulatory elements in *AeNPR1a* promoter region. The colored shapes represent different cis-elements as indicated. The scale on top of the bar represents the distance relative to the translational start condon (+1). TSS, transcription start site.

The 2000bp 5′-ﬂanking region of the *AeNPR1a* gene was isolated by chromosome walking. Meanwhile, the sequence was compared with the promoter of *AcNPR1a* in the *A. chinensis* genome database, which showed that there were many differences between the two sequences. Sequence alignment of the promoter and the *AeNPR1a* 5’-UTR region showed that the transcription start site (TSS) was located at −239 bp upstream of the ATG codon (**Figure 5**). Bioinformatic analysis revealed a putative TATA box and two CAAT box were located at the −335 and −415/−435 regions, respectively. Four W-box, which is recognized specifically by SA-induced WRKY DNA binding proteins, were identified at −126, −762, −1,459, and −1664 positions. Two potential RAV1AAT elements and a TC-rich repeats that participate in defense and stress responses were located at positions −499, −1,412, and −687. Two 5′ UTR pyrimidine-rich stretches existed in the −149 to −95 regions, which conferred high transcription. Furthermore, we also noted the presence of several cis-acting elements associated with phytohormone and abiotic stress regulation such as TCA-element, TGACG-motif, AuxRR-core, GARE-motif, MYC, and LTR (**Figure 5**), which are known involved in SA, MeJA, auxin, gibberellin, dehydration, and low temperature-associated responses, respectively.

### Expression Analysis of AeNPR1a in Different Tissues and in Response to Hormone Treatments and Psa Infection

**Figure 6:**
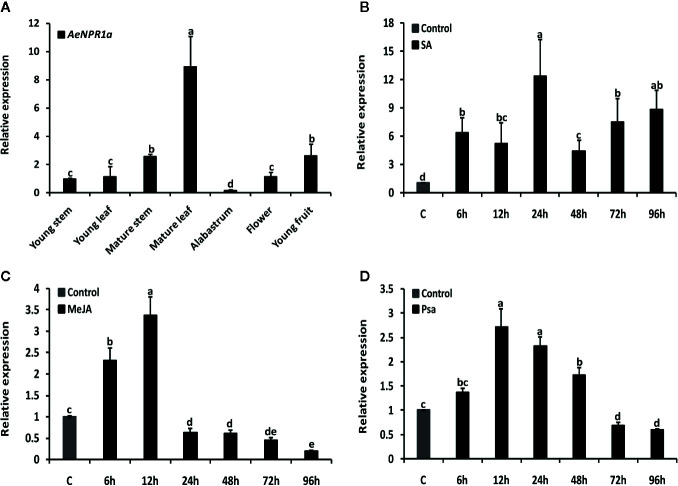
Expression patterns of *AeNPR1a* in *A. eriantha*. **(A)** Expression profiles of *AeNPR1a* in different tissues. The expression in different tissues was calibrated using expression in the young stem. **(B–D)** Expression analyses of *AeNPR1a* in leaves at various time points after SA, MeJA treatment, and Psa infection, respectively. *β-actin* was used to normalize the samples. The gene expression level at each time point is shown as relative to the mock, which was set to 1. Data represent the mean ± standard deviation (SD) of three biological replicates. Bars with different letters are significantly different (P < 0.05) according to Duncan’s multiple tests. The experiments were repeated three times with similar results.

Quantitative real-time RT-PCR analyses were performed to examine the expression levels of *AeNPR1a* in various tissues and under different treatment conditions. As shown in **Figure 6A**, the transcripts of *AeNPR1a* were detected in almost all the tissues investigated, while the levels of expression varied considerably among different tissues. The highest levels of *AeNPR1a* expression were observed in the mature leaf (8.9-fold, compared the levels in the young stem), followed by young fruit and mature stems (2.5-fold), and the lowest expression levels were detected in the alabastrum (**Figure 6A**).

It has been previously reported that exogenous plant defense molecules, such as SA, JA, and so on, can induce expression of *NPR1* or its homologous genes, to activate plant disease resistance responses (Kunkel and Brooks, 2002; Ali et al., 2017; Neeley et al., 2019). Here, the temporal expression profiles of *AeNPR1a* in leaves following treatment with SA, MeJA as well as infection with Psa, were analyzed by real-time qRT-PCR, respectively. As shown in **Figure 6**, when plants were treated with SA, *AeNPR1a* expression was induced significantly and had increased up to the peak level 24 h after treatment (**Figure 6B**). Upon MeJA treatment, *AeNPR1a* transcription was up-regulated after 6 h of treatment application, and the maximum expression levels were observed at 12 h, with rapid decline at later time points (**Figure 6C**). To further explore the defensive role of *AeNPR1a*, plants were inoculated with Psa pathogen and we monitored changes in *AeNPR1a* expression. After inoculation, *AeNPR1a* transcription increased slightly at 6 h, with a single peak 12 h post inoculation (**Figure 6D**).

### AeNPR1a Could Interact With AeTGA2

**Figure 7:**
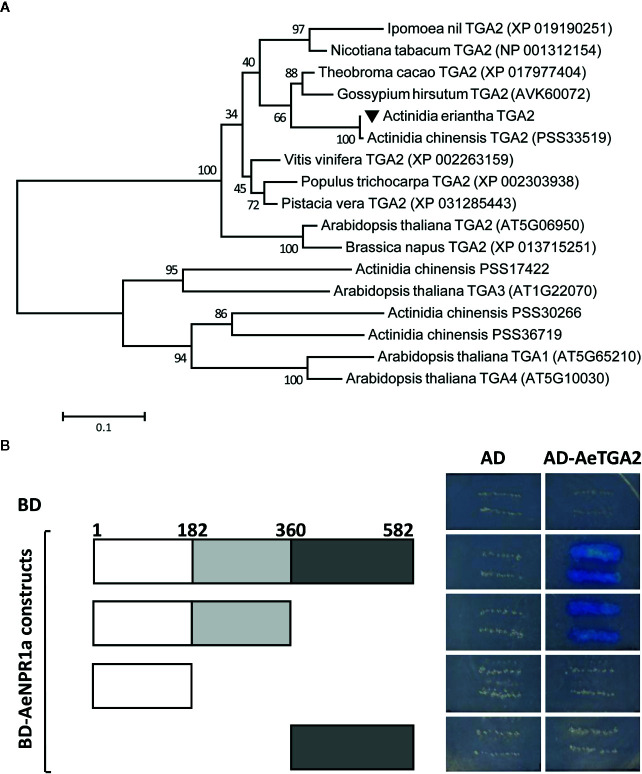
AeNPR1a interacts with AeTGA2. **(A)** Phylogenetic analysis of AeTGA2 with other TGA proteins from different plant species. The tree was generated using Maximum-Likelihood method with 1,000 bootstrap replicates. GenBank IDs of each protein sequence are attached in the brackets behind the species names. **(B)** Yeast two-hybrid assay of protein interaction between AeNPR1a and AeTGA2. Transformed yeast cells were streaked on QOD (SD/-Trp/-Leu/-His/-Ade) medium supplemented with 20 μg/ml X-α-Gal. Yeast cells co-transformed with pGBKT7 and pGADT7, pGBKT7-AeNPR1a constructs and pGADT7 were used as negative control.

Previous reports suggest that NPR1 interacts with TGA family members in *Arabidopsis* (Zhang et al., 1999). Here, the *AeTGA2* full-length cDNA sequence was obtained, phylogenetic analyses of protein sequences suggested that AeTGA2 was grouped together with other TGA2 homologs (**Figure 7A**). To further explore the potential interaction between AeNPR1a and AeTGA2 in kiwifruit, we performed yeast two-hybrid assays. The different regions of *AeNPR1a* were cloned in a pGBKT7 vector containing a DNA-binding domain to construct BD bait, and the ORF of *AeTGA2* was cloned into the pGADT7 vector with a DNA activation domain to construct AD prey (**Figure 7B**). The bait and prey plasmids were co-transformed into yeast cells and selected on QOD mediums. As shown in **Figure 7B**, both the complete AeNPR1a_(1-582)_ and the AeNPR1a_(1-360)_ truncation that included the BTB and ANK domains showed interaction with AeTGA2. However, neither the N-terminal AeNPR1a_(1-182)_ nor C-terminal AeNPR1a_(361-582)_ alone can interact with AeTGA2 (**Figure 7B**). The results indicated the interaction between AeNPR1a and AeTGA2.

### Ectopic Expression of AeNPR1a Enhanced Disease Resistance in Transgenic Tobacco

**Figure 8:**
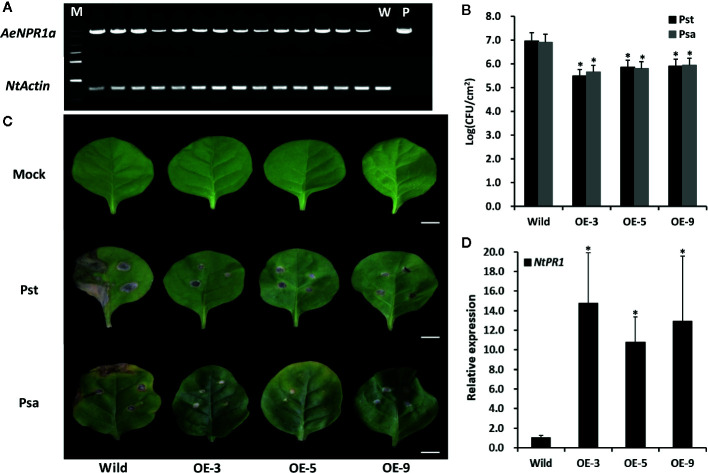
Overexpression of *AeNPR1a* in transgenic tobacco display enhanced resistance to bacterial pathogen. **(A)** RT-PCR analysis of *AeNPR1a* expression in T_2_ transgenic tobacco plants. M, DNA marker; W, the wild-type tobacco; P, the positive control (pCAMBIA plasmid). *NtActin* was used as internal control. **(B)** Growth of *Pseudomonas syringae* pv. *tomato* DC3000 (Pst) and *Pseudomonas syringae* pv. *Actinidiae* (Psa) in wild-type and *AeNPR1a* transgenic plants (OE-3, OE-5, OE-9). The leaves of 4-week-old wild-type and transgenic plants were inoculated with Pst and Psa, respectively. Bacterial population was monitored 3 days after inoculation using serial dilution. Each data point represents the mean and standard errors of three samples from three individual plants. The asterisks represent statistically significant differences between the wild-type and *AeNPR1a* transgenic plants (P < 0.05). CFU: Colony forming unit. **(C)** Disease symptoms from representative leaves of wild-type and transgenic plants at 7 days after inoculation with Pst, Psa, or 10 mM MgCl_2_ (Mock). Bars = 1 cm. **(D)** Transcript analysis of *NtPR1* gene in transgenic plants by quantitative PCR. *NtActin* was used to normalize the samples. Asterisks indicate a significant difference (Student’s t-test, P < 0.05). The results are presented from three independent experiments.

In order to assess the function of the *AeNPR1a*, we constitutively expressed *AeNPR1a* in tobacco. A total of 14 independent transgenic lines were generated by *Agrobacterium*-mediated transformation, and transgene expression was confirmed by semi-quantitative RT-PCR analysis (**Figure 8A**). These transgenic lines appeared normal in their growth and development compared to wild-type plants (**Supplementary Table S3** and **Supplementary Figure S2**). Three transgenic lines were randomly selected to generate T_2_ generation plants for further investigation.

To determine whether *AeNPR1a* over-expression confers resistance to pathogenic bacteria, the transgenic and wild-type plants were inoculated with Pst and Psa, separately. As shown in **Figure 8B**, the bacterial populations of both Pst and Psa in the wild-type leaf discs increased significantly when compared to the levels in the *AeNPR1a* transgenic lines 3 days after inoculation. Seven days after infection, wild-type leaves displayed severe necrotic lesions (**Figure 8C**). Although the *AeNPR1a* transgenic lines also exhibited disease symptoms, the lesions degree in necrotic phenotype was comparatively lower than wild-type (**Figure 8C**). Generally, the expression of *PR* genes has been considered a marker for plant disease resistance. To understand whether pathogen resistance in *AeNPR1a* transgenic lines was related to *PR* induction, the expression of *NtPR1* in the transgenic tobacco lines were analyzed. As shown in **Figure 8D**, the transcript levels of *NtPR1* in transgenic plants increased significantly relative to the levels in the wild-type. These results demonstrated that *AeNPR1a* overexpression in tobacco confers enhanced resistance to bacterial pathogens, and the resistance could be associated with increased expression of *PR* gene.

### AeNPR1a Complements Resistance to Pseudomonas syringae pv. Tomato DC3000 in Arabidopsis npr1-1 Mutant

**Figure 9:**
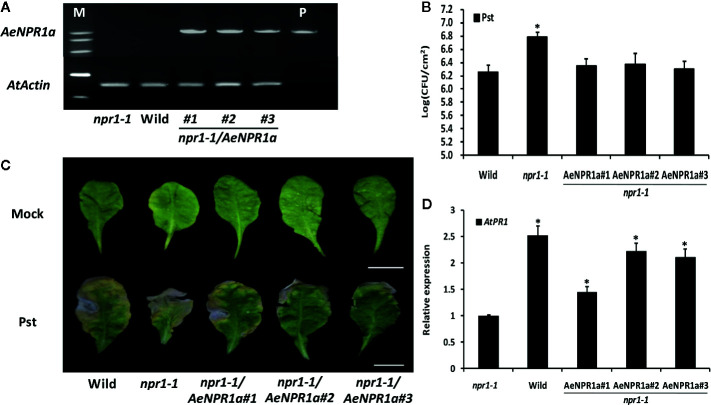
Complementation of the *Arabidopsis npr1-1* mutants by *AeNPR1a*. **(A)** RT-PCR analysis of *AeNPR1a* expression was performed with cDNA prepared from leaves of *npr1-1*, wild type and transgenic *npr1-1* mutant lines. M, DNA marker; P, the positive control (pCAMBIA plasmid). *AtActin* was used as internal control. **(B)** Bacterial growth of *Pseudomonas syringae* pv. *tomato* DC3000 (Pst) on leaves of 4-week-old wild-type, *npr1-1*, and three individual *npr1-1/AeNPR1a* transgenic plants. Samples were taken from infected leaves 3 days after inoculation to quantify colony-forming units (CFU). Data represent the means ± standard errors of three biological replicates, and each containing three leaf disks from three individual plants. The asterisk indicates statistically significant differences from the wild-type (P < 0.05). **(C)** Symptoms on leaves infected with Pst or mock (10 mM MgCl_2_) at 7 days post-inoculation. Bars = 1 cm. **(D)** Analysis of *AtPR1* gene expression in *npr1-1*, wild type and transgenic plants. Values are shown as mean ± standard deviation (SD) for three replicates, and representative results are presented from three independent experiments. Asterisks indicate a significant difference (Student’s t-test, P < 0.05%).

To further investigate if *AeNPR1a* has functions similar to *Arabidopsis NPR1*, the *35S::AeNPR1a* construct was transformed into the *Arabidopsis npr1-1* mutant for stable transformants by *Agrobacterium*-mediated transformation (**Figure 9A**). Three independent T_3_ transgenic lines expressing *AeNPR1a* and *npr1-1* mutant, along with wild-type plants, were infected with bacterial Pst suspended in 10 mM MgCl_2_, or 10 mM MgCl_2_ alone as a mock. Three days after inoculation, the population of Pst on infected leaves was measured to quantify the disease symptoms. As shown in **Figure 9B**, the levels of bacterial growth in infected *npr1-1* mutants were more than 3 times the levels in the wild-type. Three transgenic lines expressing *AeNPR1a* had significant decreases in bacterial growth when compared to the levels in *npr1-1* mutant (**Figure 9B**). After 7 days of inoculation, the leaves of *npr1-1* mutants showed severe necrotic lesions, while such symptoms in transgenic plants were restricted (**Figure 9C**). In addition, the over-expression of *AeNPR1a* in the *npr1-1* mutant significantly elevated the *AtPR1* expression (**Figure 9D**). Overall, the observations above indicated that *AeNPR1a* can functionally complement the basal resistance to Pst in the *npr1-1* mutant plants.

## Discussion

Plants employ diverse signal transduction pathways to defend themselves against infection by pathogens. SAR is a one of the major defense pathways that can confer long-lasting resistance in plants, and SAR establishment is mediated predominantly by *NPR1* in *Arabidopsis* (Gao et al., 2015; Zhang et al., 2019). It has been revealed that *NPR1* is involved in various biotic defense or abiotic stress signaling pathways (Cao et al., 1998; Wu et al., 2012; Withers and Dong, 2016). Although homologs of the *NPR1* gene have been isolated and characterized in several plant species, our understanding of the function of *NPR1* homologs in kiwifruit remains poor. In the present study, a novel *NPR1*-like gene (*AeNPR1a*) from kiwifruit was characterized. The intron/exon organization of *AeNPR1a* exhibited structures similar to *NPR1* in *Arabidopsis*, as well as in other species. The derived amino acid sequence of AeNPR1a shows 52.86% identity to *Arabidopsis* NPR1, and they shared typical features, such as a BTB/POZ domain and an ankyrin repeat, which are highly conserved among all NPR1-type proteins of monocotyledonous and dicotyledonous plants (Cao et al., 1997; Silva et al., 2018; Backer et al., 2019). These domains are responsible for the co-activation of TGA transcription factors and protein-protein interactions (Rochon et al., 2006; Ding et al., 2018). The cysteine residues, Cys^82^, Cys^150^, Cys^155^, Cys^160^, and Cys^216^ are highly conserved among all the sequences, and they could be involved in the oligomer-monomer transition of NPR1 or NPR1-like proteins (Mou et al., 2003). It has been demonstrated that the S-nitrosylation of NPR1 at Cys^156^ could facilitate its oligomerization in *Arabidopsis* (Tada et al., 2008). Cys^156^ in AeNPR1a was altered to a Serine residue. Previous studies have reported that although Cys^156^ in the GmNPR1-1 protein of soybean is substituted, GmNPR1-1 still complements NPR1 function in the *npr1-1* mutant, which might involve other residues, to maintain GmNPR1-1 protein homeostasis (Sandhu et al., 2009). This means that some other conserved residues of AeNPR1a might also play critical roles in defense regulation in kiwifruit. In addition, the AeNPR1a protein also has a potential nuclear localization motif in the C-terminus (**Figure 1**). The data suggest that the AeNPR1a protein shared some common characteristics with NPR1 from other plants.

Phylogenetic analysis revealed that NPR1-like proteins in kiwifruit were clustered in different clades; the AeNPR1a was closer to VvNPR1 of *Vitis vinifera* and grouped in the same clade as AtNPR1, which is essential for SAR establishment (Cao et al., 1997; Dong, 2004). It have been reported that AtNPR2, as a paralog of AtNPR1, can complement *npr1* mutants and play a role in SA perception (Castelló et al., 2018). The phylogenic relation of AeNPR1a and AeNPR1b implied that AeNPR1b might also act as component of the SA signal pathway in kiwifruit. According to previous studies, nuclear localization of NPR1 is essential for its function, which is directed by the NLS in the C-terminus (Kinkema et al., 2000). In *Arabidopsis*, NPR1 is mainly localized in the cytoplasm in the form of oligomers in conditions with no biotic stress. Upon SA induction, monomeric NPR1 is released from the oligomers and transported into the nucleus (Mou et al., 2003; Maier et al., 2011). However, in the present study, unlike AtNPR1, which also showed a small amount of fluorescence in the plasma membrane of onion epidermal cells, the transiently expressed AeNPR1a-EGFP fusion protein was predominantly located in the nucleus of *Arabidopsis* protoplast cells and onion epidermal cells (**Figure 4** and **Supplementary Figure S1**). Differences in species or key amino acid residues could have promoted the translocation of AeNPR1a into the nucleus. Consistently, constitutive nuclear localization has also been reported by transient expression of StoNPR1 and VvNPR1, even in the absence of SA induction (Le Henanff et al., 2009; Jue et al., 2014). The results suggest that AeNPR1a is primarily localized in the nucleus under non-inductive condition.

The specific gene expression is mainly driven by the promoter. To reveal the regulatory mechanism of *AeNPR1a* expression, a 2.0 Kb *AeNPR1a* promoter was isolated, and several cis-acting regulatory elements, including phytohormone and defense responsive elements, were identified, which suggested that *AeNPR1a* could be regulated by plant hormones and stress factors. The expression patterns of *AeNPR1a* were analyzed by qRT-PCR, and it was expressed constitutively in different tissues, particularly in mature leaves. The observed expression patterns differed from the high levels of expression of *NPR1* homologs in roots and stems of avocado and soybean, respectively (Sandhu et al., 2009; Backer et al., 2015). We also present evidence that *AeNPR1a* transcript is expressed in reproductive tissues. Previous studies have demonstrated that *NPR1* expression can be induced by exogenous signaling molecules, such as SA, JA, and ethylene, to activate plant defense responses and confer resistance to pathogens (Kunkel and Brooks, 2002; Leon-Reyes et al., 2009; Wang et al., 2017). *AeNPR1a* transcription was induced not only by treatment with SA or MeJA but also by pathogen infection, which may be associated with the presence of phytohormone and defense responsive elements in the promoter. In contrast, the expression of *BjNPR1* in mustard was not altered after MeJA treatment (Ali et al., 2017). The discrepancy could be attributed to variation of the mechanism of signal molecules in different species (Kunkel and Brooks, 2002; Caarls et al., 2015). In addition, *AeNPR1a* expression was induced rapidly by each treatment in 24 h, suggesting that the gene might participate in the early stages of defense responses to treatment with exogenous signaling molecules and pathogen infection. Similar results were also observed in rice, lily, and *Mimulus lewisii* (Yuan et al., 2007; Wang et al., 2017; He and Shi, 2018). In *Arabidopsis*, the interaction between NPR1 and TGA2 family proteins is known to be important for the activation of defense genes, and conferring resistance to secondary infection (Fan and Dong, 2002; Silva et al., 2018). Therefore, the potential interaction between AeNPR1a and AeTGA2 was assessed using the yeast two-hybrid system. Our results not only support the existence of an interaction between the two proteins but also highlighted some regions of AeNPR1a protein that are critical for the interaction. However, whether AeNPR1a-AeTGA2 interaction regulates *PR* genes transcription to trigger SAR responses in kiwifruit still requires verification.

Numerous molecular studies have shown that *NPR1* or *NPR1*-like genes confer resistance to bacterial and fungal pathogens in various transgenic plants, and the resistance was associated with *PR* expression (Cao et al., 1998; Le Henanff et al., 2011; Zhang et al., 2012; Ali et al., 2017). Transgenic citrus plants expressing *AtNPR1* exhibited significant up-regulation of *PR1* expression and enhanced resistance to citrus canker and Huanglongbing (Zhang et al., 2010; Robertson et al., 2018). Ectopic expression of *Malus hupehensis MhNPR1* in tobacco induced the transcription of *PR* genes and facilitated salt and osmotic stress tolerance, in addition to enhanced resistance to *Botrytis cinerea* (Zhang et al., 2012; Zhang et al., 2014). Similarly, in the present study, the over-expression of *AeNPR1a* in transgenic tobacco plants triggered disease resistance against both Pst and Psa bacterial pathogens, as transgenic lines had reduced pathogen proliferation and limited disease spread to non-infected leaf parts. It has been reported that over-expression *AtNPR1* in transgenic strawberry plants improved resistance to pathogens, but the plants displayed numerous undesirable traits with regard to growth and development, such as decreased plant height, canopy density, and defective fruits (Silva et al., 2015). The transgenic *Arabidopsis* plants that express mulberry *MuNPR1* produced an early flowering phenotype (Xu et al., 2019). However, *AeNPR1a* transgenic tobacco exhibited normal phenotypes and did not exhibit any detrimental morphological traits under our experimental conditions, and similar results have been reported in cotton, citrus, and mustard (Parkhi et al., 2010; Dutt et al., 2015; Ali et al., 2017). Notably, the transcript levels of the SAR marker *NtPR1* were increased significantly in transgenic tobacco lines without an exogenous inducer. A possible explanation for the phenomenon is *AeNPR1a* transcription in transgenic lines achieved a certain threshold that is required for *NtPR1* activation, or biotic stress from T-DNA insertions might persist in transgenic tobacco plants.

The *npr1* mutant *Arabidopsis* plants fail to induce SAR in response to various inducing agents and display increased susceptibility to pathogen infections (Cao et al., 1994). In the present study, the effects of complementation of the *Arabidopsis npr1-1* mutant with *AeNPR1a* were analyzed. Over-expression of *AeNPR1a* in the *npr1-1* mutant increased resistance to Pst infection, which is consistent with previous observations in other plant species, including rice and *Gladiolus* (Yuan et al., 2007; Zhong et al., 2015). Consequently, we conclude that *AeNPR1a* is orthologous to *Arabidopsis NPR1* and might participate in mechanisms of defense against pathogens in kiwifruit.

In summary, the results of the presented study demonstrate that *AeNPR1a* can be induced by phytohormones and Psa infection. We provide evidence that AeNPR1a can interact with AeTGA2. In addition, ectopic expression of *AeNPR1a* in transgenic tobacco and *Arabidopsis* *npr1-1* mutant confers resistance against bacterial pathogens, based on the observed differences in the degrees of severity of disease symptoms between transformed and untransformed lines. Our results could facilitate efforts to enhance disease resistance in kiwifruit through biotechnological approaches that target endogenous defense mechanisms. In future research, over-expression or knockout of *AeNPR1a* could be carried out in kiwifruit, and transgenic plants would subsequently be challenged under biotic and abiotic stresses. In addition, the promoter function of the gene will be characterized to further elucidate the defense pathways and networks mediated by *AeNPR1a*. The finding of such studies would facilitate the genetic improvement of kiwifruit and lead to the development of more resistant resources.

## Data Availability Statement

The datasets presented in this study can be found in online repositories. The names of the repository/repositories and accession number(s) can be found in the article/**Supplementary Material**.

## Author Contributions

L-MS and J-BF participated in research design. L-MS carried out most of the experiments and wrote the manuscript. L-MS and J-BF were responsible for generating the data and for interpretation of the results. M-ML performed the real-time PCR experiments. X-JQ and J-YC contributed in the statistical analyses. MZ carried out genetic transformation. All authors contributed to the article and approved the submitted version.

## Funding

This work was financially supported by the National Key Research and Development Program of China (2019YFD1000200), the National Natural Science Foundation of China (grant no. 31701778), the Agricultural Science and Technology Innovation Program (ASTIP) (CAAS-ASTIP-2020-ZFRI-04), and Modern agricultural industry technology system of Henan province (S2014-11).

## Conflict of Interest

The authors declare that the research was conducted in the absence of any commercial or financial relationships that could be construed as a potential conflict of interest.
