# Supplementary material for: Molecular Cloning and Functional Analysis of the NPR1 Homolog in Kiwifruit (Actinidia eriantha)
Source: Front Plant Sci. 2020 Sep 16;11:551201. doi: 10.3389/fpls.2020.551201 (PMC7524898; doi:10.3389/fpls.2020.551201)
Supplement: Supplementary file 1 [file Table_1.doc]

Supplementary Material

# 1 Supplementary Tables

| **Supplementary Table S1. The primers used in this study** | | |
| --- | --- | --- |
| The primers for gene amplication: | | |
| RACE primers | Forward(NPR-F1): | 5`-GGGCTCTGGATTCTGATGATGTTG-3` |
|  | Forward(NPR-F2): | 5`-GAGGAAGGGAAAGCATCTCCCAA-3` |
|  | Reverse(NPR-R1): | 5`-GACAAGATGTTGGGGCAAGGC-3` |
|  | Reverse(NPR-R2): | 5`-CTCTGATAAAGCGCAACCAGTTC-3` |
| *AeNPR1a* | Forward(NPR1-01): | 5`-TCTCACTTTTAGCATCCCCTCT-3` |
|  | Reverse(NPR1-02): | 5`-TCACCCAAATCATCAATCCA-3` |
| The primers for walking: | | |
| *AeNPR1a* | NP-01: | 5`-CAGAGAGGTTAGTTTAGTGAT-3` |
|  | NP-02: | 5`-GCTGAAGGTTGATGACATAAGCTA-3` |
|  | NP-03: | 5`-CCAGCTTGATGACGGATTG-3` |
|  | NP-04: | 5`-GGTCTAGTTTGGACTTAGCTAA-3` |
| The primers for Real-time quantitatively RT-PCR: | | |
| *AeNPR1a* | Forward: | 5`-CAAGAACCGTGGAATTGGGA-3` |
|  | Reverse: | 5`-TATCCTCGTTGAACGCCTTG-3` |
| *β-actin* | Forward: | 5`-AAGCCCGATCCAGCAATA-3` |
|  | Reverse: | 5`-GCCCTTTACGCCCAGTCA-3` |
| *NtPR1* | Forward: | 5`-ATTGCCTTCATTTCTTCTTGTC-3` |
|  | Reverse: | 5`-TATGGGCATCCAAATAGTCTTG-3` |
| *NtActin* | Forward: | 5`-AGATGTTCCGTCGTGTCAGTG-3` |
|  | Reverse: | 5`-TGCTTCCTCTTCATCCTCATATCC-3` |
| *AtPR1* | Forward: | 5`-GCTCTTGTTCTTCCCTCGAA-3` |
|  | Reverse: | 5`-GCCTCTTAGTTGTTCTGCGTAG-3` |
| *AtActin* | Forward: | 5`-GCACCCTGTTCTTCTTACCG-3` |
|  | Reverse: | 5`-AACCCTCGTAGATTGGCACA-3` |
| The primers for expression vector construct: | | |
| *AeNPR1a* | Forward(NPR1-03): | 5`-AGATCTTCTCACTTTTAGCATCCCCTCT-3` |
|  | Reverse(NPR1-04): | 5`-GGTAACCTCACCCAAATCATCAATCCA-3` |
| The primers for yeast two-hybrid assay: | | |
| *AeNPR1aBD1* | Forward: | 5`-CATATGGATACTCGAACCGCCTTC-3` |
|  | Reverse: | 5`-GGATCCCTACTTCTTGAAGATGAG-3` |
| *AeNPR1aBD2* | Forward: | 5`-CATATGGATACTCGAACCGCCTTC-3` |
|  | Reverse: | 5`-GGATCCTGAAATCTGAAGTGCTTT-3` |
| *AeNPR1aBD3* | Forward: | 5`-CATATGGATACTCGAACCGCCTTC-3` |
|  | Reverse: | 5`-GGATCCAAGAATATCCAGTAGGCG-3` |
| *AeNPR1aBD4* | Forward: | 5`-CATATGAAGAGGCTCACCAGAGCT |
|  | Reverse: | 5`-GGATCCCTACTTCTTGAAGATGAG-3` |

| **Supplementary Table S2. NPR1-like protein sequences from other plant species for phylogram construction** | | | |
| --- | --- | --- | --- |
| **Identifier** | **Accession number** | **Species** | **Lineage** |
| AcNPR1a | PSS20797 | *Actinidia chinensis* | Dicotyledon |
| AcNPR1b | PSS36420 | *A. chinensis* | Dicotyledon |
| AcPSR84640 | PSR84640 | *A. chinensis* | Dicotyledon |
| AcPSR96484 | PSR96484 | *A. chinensis* | Dicotyledon |
| AcPSS11132 | PSS11132 | *A. chinensis* | Dicotyledon |
| AeNPR1b | 01g14520 | *Actinidia eriantha* | Dicotyledon |
| Ae23g12490 | 23g12490 | *A. eriantha* | Dicotyledon |
| Ae14g01000 | 14g01000 | *A. eriantha* | Dicotyledon |
| AtNPR1 | [AT1G64280](https://www.arabidopsis.org/servlets/TairObject?type=locus&name=AT1G64280) | *Arabidopsis thaliana* | Dicotyledon |
| AtNPR2 | AT4G26120 | *A. thaliana* | Dicotyledon |
| AtNPR3 | AT5G45110 | *A. thaliana* | Dicotyledon |
| AtNPR4 | [AT4G19660](https://www.arabidopsis.org/servlets/TairObject?type=locus&name=AT4G19660) | *A. thaliana* | Dicotyledon |
| TcNPR1 | ADI24348 | *Theobroma cacao* | Dicotyledon |
| TcNPR3 | AGC39275 | *T. cacao* | Dicotyledon |
| TcNPR4 | XP_007024872 | *T. cacao* | Dicotyledon |
| GhNPR1 | ABC54558 | *Gossypium hirsutum* | Dicotyledon |
| BjNPR1 | ABC94642 | *Brassica juncea* | Dicotyledon |
| CcNPR1 | XP_006451500 | *Citrus clementina* | Dicotyledon |
| RcNPR1 | XP_002514127 | *Ricinus communis* | Dicotyledon |
| PtNPR1 | XP_002308281 | *Populus trichocarpa* | Dicotyledon |
| PeNPR1 | XP_011003334 | *Populus euphratica* | Dicotyledon |
| PeNPR3 | XP_011040366 | *P. euphratica* | Dicotyledon |
| GmNPR1 | XP_003534926 | *Glycine max* | Dicotyledon |
| GmNPR1-1 | NP_001238658 | *G. max* | Dicotyledon |
| GmNPR1-2 | NP_001238674 | *G. max* | Dicotyledon |
| MtNPR1 | XP_003595225 | *Medicago truncatula* | Dicotyledon |
| MtNPR1-1 | XP_003594464 | *M. truncatula* | Dicotyledon |
| FvNPR1 | XP_004288961 | *Fragaria vesca* | Dicotyledon |
| FvNPR3 | XP_004293734 | *F. vesca* | Dicotyledon |
| PmNPR1 | XP_008242373 | *Prunus mume* | Dicotyledon |
| PmNPR3 | XP_008225741 | *P. mume* | Dicotyledon |
| PbNPR1 | XP_009361630 | *Pyrus* × *bretschneideri* | Dicotyledon |
| MdNPR1 | XP_008391028 | *Malus* × *domestica* | Dicotyledon |
| MdNPR3 | NP_001280793 | *M.* *domestica* | Dicotyledon |
| VvNPR1 | XP_002281475 | *Vitis vinifera* | Dicotyledon |
| VvNPR3 | XP_002274045 | *V. vinifera* | Dicotyledon |
| SinNPR1 | XP_011078001 | *Sesamum indicum* | Dicotyledon |
| IbNPR1 | ABM64782 | *Ipomoea batatas* | Dicotyledon |
| NtNPR1 | AF480488 | *Nicotiana tabacum* | Dicotyledon |
| CaNPR1 | ABG38308 | *Capsicum annuum* | Dicotyledon |
| StNPR1 | XP_006357709 | *Solanum tuberosum* | Dicotyledon |
| SlNIM1 | NP_001234558 | *Solanum lycopersicum* | Dicotyledon |
| CsNPR1 | XP_010418483 | *Camelina sativa* | Dicotyledon |
| CsNPR3 | XP_010494583 | *C. sativa* | Dicotyledon |
| EgNPR1 | XP_010915286 | *Elaeis guineensis* | Monocotyledon |
| EgNPR3 | XP_010908601 | *E. guineensis* | Monocotyledon |
| PdNPR1 | XP_008782513 | *Phoenix dactylifera* | Monocotyledon |
| PdNPR3 | XP_008806697 | *P. dactylifera* | Monocotyledon |
| LhSorNPR1 | APG55777 | *Lilium hybrid* | Monocotyledon |
| GlNPR1 | AIM54370 | *Gladiolus hybrid* | Monocotyledon |
| ZmNPR1 | DAA52994 | *Zea mays* | Monocotyledon |
| SitNPR1 | XP_004968514 | *Setaria italica* | Monocotyledon |
| OsNPR1 | AAX18700 | *Oryza sativa* | Monocotyledon |
| BdNPR1 | XP_003564857 | *Brachypodium distachyon* | Monocotyledon |
| HvNPR1 | CAJ19095 | *Hordeum vulgare* | Monocotyledon |
| TdNPR1 | AGH18701 | *Triticum durum* | Monocotyledon |

| **Supplementary Table S3. Phenotypic analysis of wild type and *AeNPR1a* transgenic tobacco did not show significant variations in terms of flowering time and stem length at flowering** | | |
| --- | --- | --- |
| **Type** | **Flowering days from transplant** | **Stem length (cm)** |
| Wild | 104.25±2.05^a^ | 57.21±3.61 ^a^ |
| OE3 | 102.88±3.40^a^ | 59.50±2.52 ^a^ |
| OE5 | 103.75±2.76^a^ | 55.44±2.21 ^a^ |
| OE9 | 105.65±4.17^a^ | 57.81±3.03 ^a^ |
| Note: values are the arithmetical mean of repetitions ± SD. Significant differences by Duncan’s multiple range test (P <0.05) are indicated by different letters. | | |

# 2 Supplementary Figures


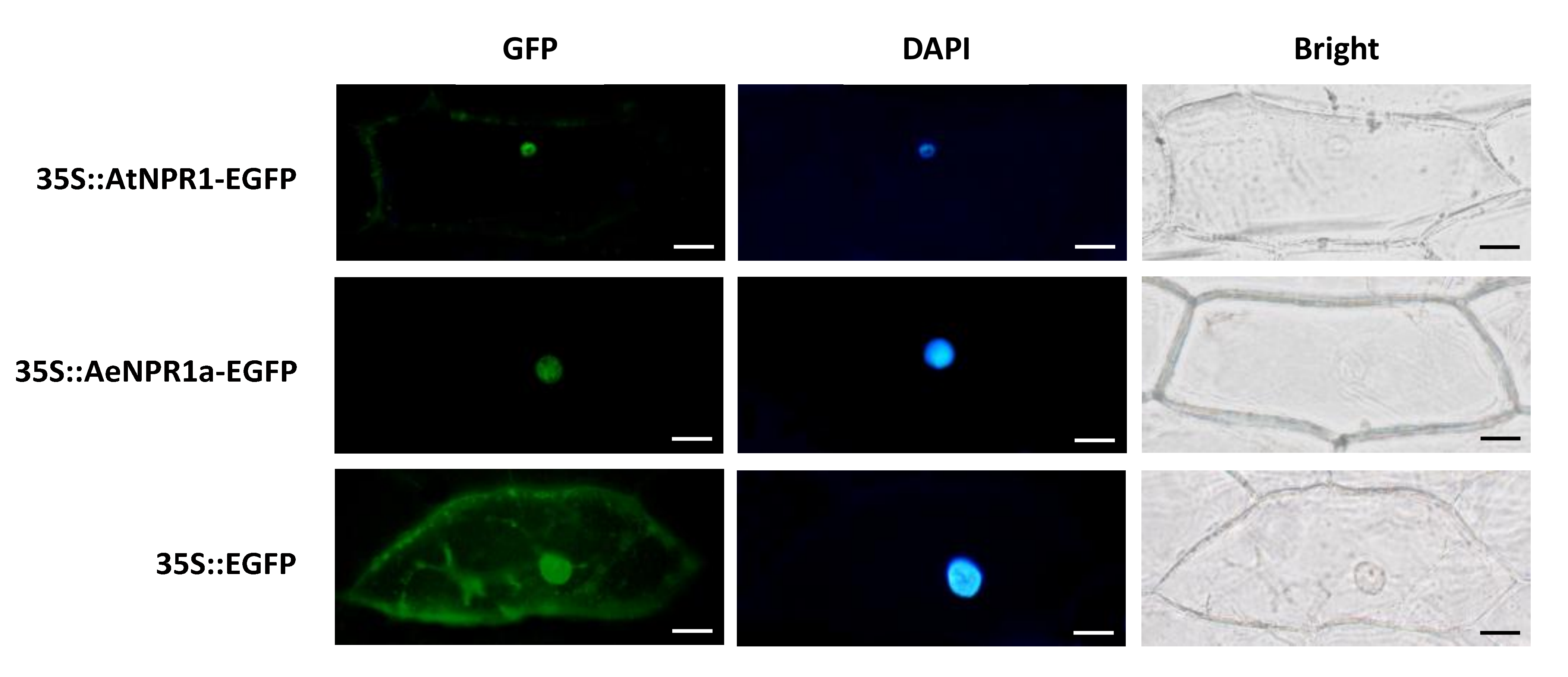


Supplementary Figure S1. Subcellular localization of AeNPR1a protein in onion epidermal cells. The constructs of *35S::AtNPR1-EGFP*, *35S::AeNPR1a-EGFP* and *35S::EGFP* were transformed into onion epidermal cells. The ﬂuorescence signals were examined by a laser confocal scanning microscope. Nuclei of the onion cells were stained with DAPI. Scale bars=50mm.


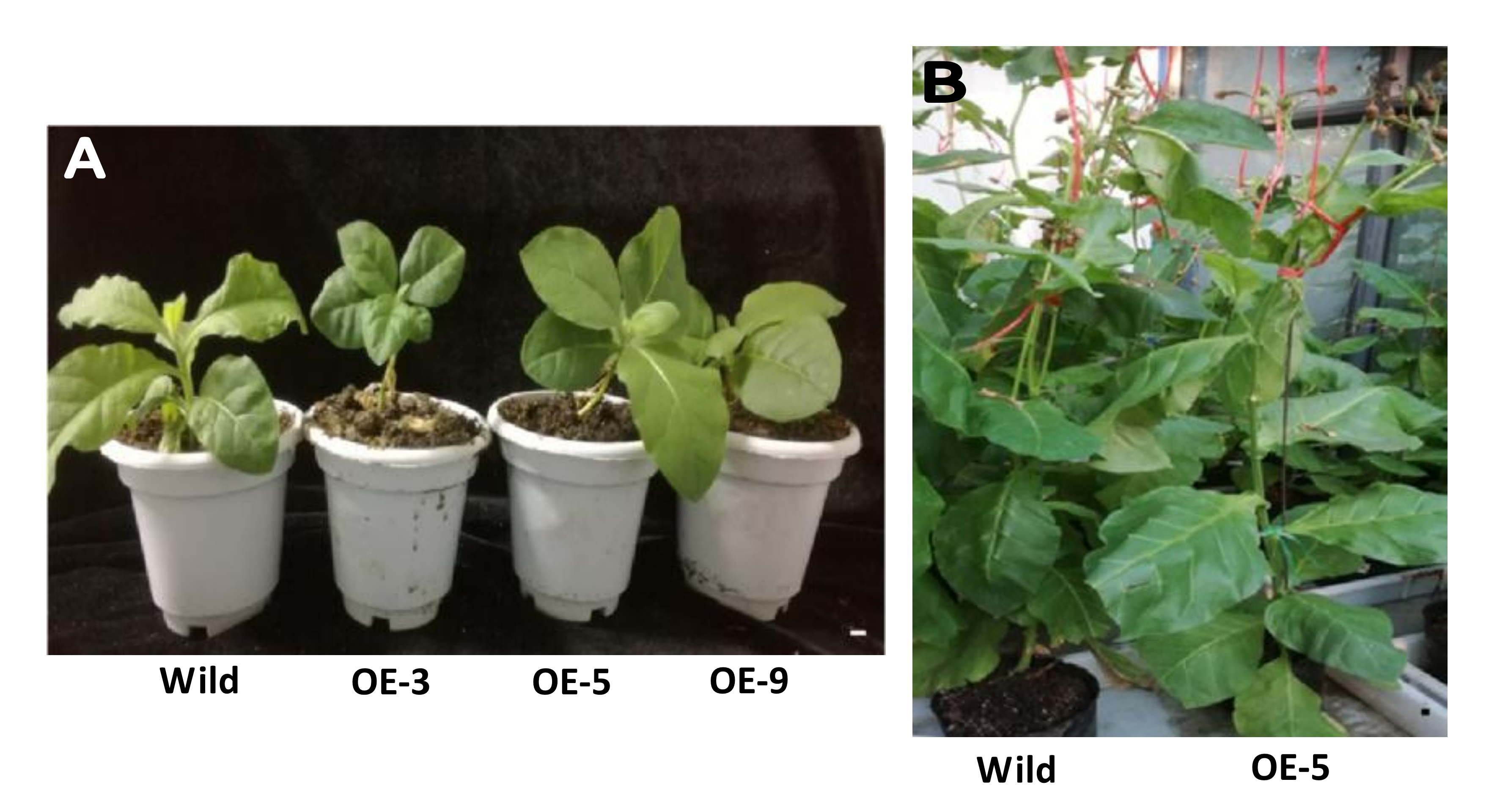


Supplementary Figure S2. Phenotypes of wild type and *AeNPR1a* transgenic tobacco after transplanting (A) and during flowering and fruiting (B). Bars=1cm.


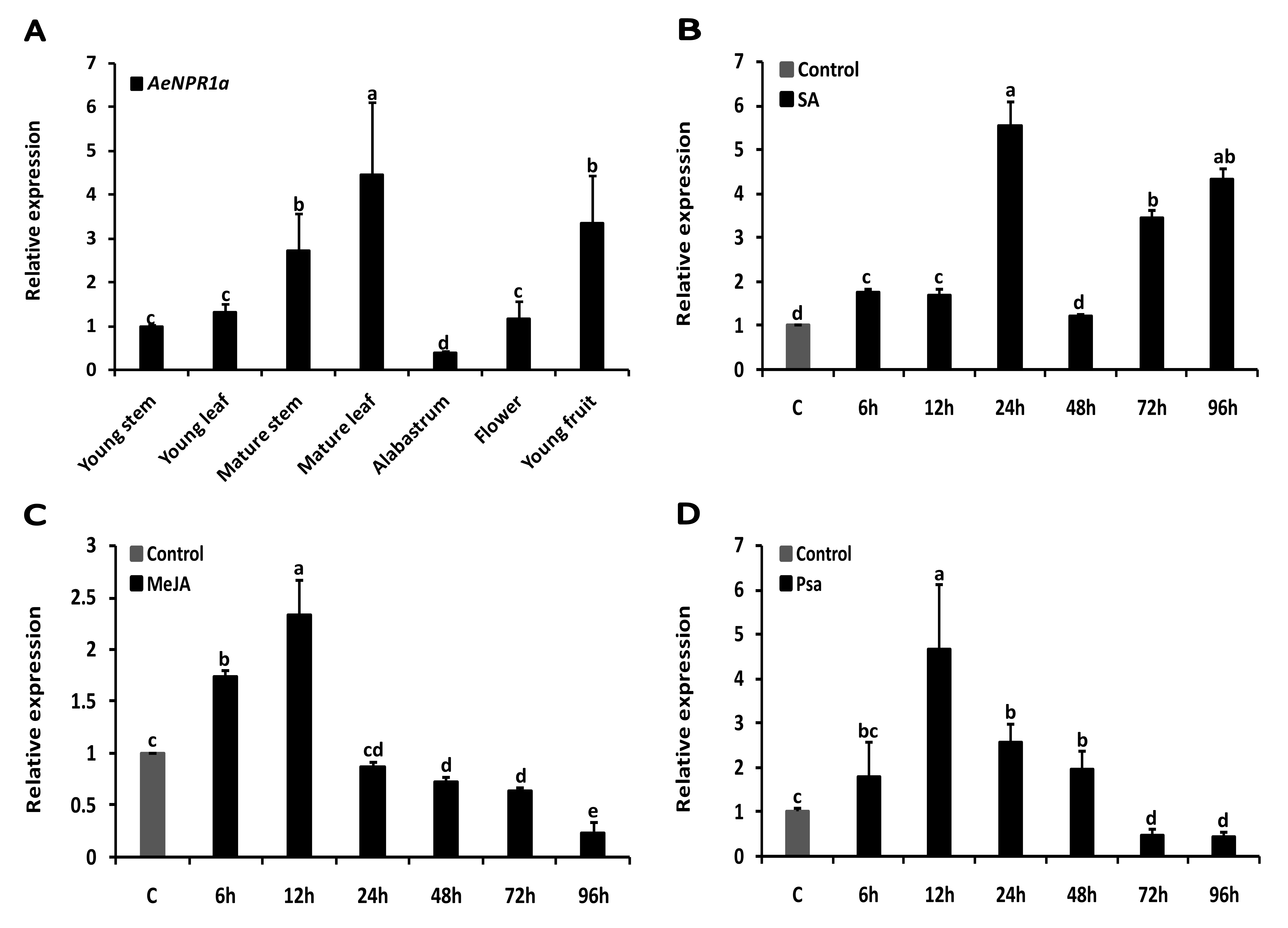


Supplementary Figure S3. Expression patterns of *AeNPR1a* in *A. eriantha*. (A) Expression profiles of *AeNPR1a* in different tissues. The expression in different tissues was calibrated using expression in the young stem. (B-D) Expression analyses of *AeNPR1a* in leaves at various time points after SA, MeJA treatment, and Psa infection, respectively. *β-actin* transcript levels were used to normalize the samples. The gene expression level at each time point is shown as relative to the mock, which was set to 1. Data represent the mean ± standard deviation (SD) of three biological replicates. Bars with different letters are significantly different (P<0.05) according to Duncan’s multiple tests.
